# Psychiatric disorders among children and adolescents with active epilepsy in southwestern Uganda

**DOI:** 10.11604/pamj-oh.2020.3.9.25146

**Published:** 2020-10-27

**Authors:** Joseph Kirabira, Alice Lam, Bashir Ssuna, Godfrey Zari Rukundo

**Affiliations:** 1 Department of Psychiatry, Faculty of Medicine, Mbarara University of Science and Technology, PO Box 1410, Mbarara, Uganda; 2 Department of Mental Health, Gulu University, PO Box 166, Gulu, Uganda; 3 Department of Mental Health and Psychiatry, Kampala International University, Western Campus, PO Box 71, Bushenyi, Uganda; 4 Department of Neurology, Massachusetts General Hospital and Harvard Medical School, Boston, MA, USA; 5 Department of Epidemiology and Biostatistics, Makerere College of Health Sciences (MakCHS), Kampala, Uganda

## Abstract

**Introduction:**

the study aimed to determine the prevalence of emotional, behavioral, developmental and psychosis related disorders among children and adolescents with active epilepsy aged 5 to 18 years in southwestern Uganda.

**Methods:**

we conducted a cross sectional study at one big urban hospital, two rural health centers and one rural special needs school. The disorders were assessed using an adapted parent version of the Child and Adolescent Symptom Inventory-5 (CASI-5).

**Results:**

one hundred and sixty-one participants were assessed, and 93 (57.8%) had at least one psychiatric disorder. Developmental disorders were the most prevalent at 39.8% (95%CI 32.11 – 47.39), followed by emotional disorders, 30.4% (95%CI 23.25–37.62), behavioral disorders, 7.5% (95%CI 3.35–11.55) and psychosis related disorders, 6.2% (95%CI 2.44 – 9.98). Thirty-nine participants (24.2%) had at least two psychiatric disorders. Developmental disorders were associated with younger age (aOR=0.86, p=0.001) and having epilepsy-related physical injuries and deformities (aOR=2.36, p=0.036). Emotional disorders (aOR=1.13, p=0.007) and psychosis related disorders (aOR=1.44, p=0.007) were associated with increasing age, whereas a family history of epilepsy was protective (aOR=0.22, p=0.042).

**Conclusion:**

psychiatric disorders were highly prevalent among children and adolescents with epilepsy in southwestern Uganda; highlighting the need to integrate screening and management of these disorders into routine epilepsy care.

## Introduction

Epilepsy is a neurological disorder with a high incidence in childhood and having a lifetime prevalence of 5.8–15.4 per 1000 people [1–3]. According to literature from developed countries, some psychiatric disorders are associated with epilepsy [4–9] possibly due to the pathophysiology and chronic nature of the condition. Psychiatric disorders may persist even after treatment of epilepsy [10] especially among children and adolescents. When not assessed and treated, psychiatric disorders may significantly affect the quality of life and functioning of people with epilepsy [11–14]. Previous studies mainly conducted in the developed world have reported socio-demographic and clinical factors that are associated with psychiatric co-morbidities in epilepsy, including age of onset; type, frequency, severity, and duration of seizures; anti-epileptic drug use; other chronic medication use; and medication side effects [5,6,15]. There is paucity of data about these comorbid disorders in the developing world, including Uganda, which carries the world’s largest burden of epilepsy. This cross-sectional study focused on understanding the psychiatric co-morbidities and associated risk factors in children and adolescents with epilepsy in southwestern Uganda.

## Methods

### Study sites:

this study was carried out in southwestern Uganda at Mbarara Regional Referral Hospital (MRRH) psychiatry ward and three other community outreach sites, including St Joseph’s Rubindi Health Center III (RHC III), Mushanga Health Centre III (MHC III) and Tukore Invalids Primary School (TIPS). Southwestern Uganda consists of Bantu (majority) people with multiple religious beliefs that include Christianity, Islam and African traditional belief systems. The economic activities carried out in this region include animal rearing, crop farming, minimal fishing, and trading in both agriculture and non-agriculture products mainly in the urban/semi urban centers. Mbarara district has approximately 113,164 households with a projected total population of about 474,144 according to the National Population and Housing Census in 2014. MRRH is the biggest public hospital in the southwestern region of Uganda, located in Kamukuzi division, Mbarara municipality, Mbarara district, approximately 265 km from Kampala city. It was founded in 1940 and serves as a referral center for all other health units in the health region. It is also a teaching hospital for Mbarara University of Science and Technology (MUST) and many other medical institutions in the region. It offers general, specialized, consultant and emergency medical service with 350 beds. The psychiatry ward has 40 beds and admits over 1000 patients each year. The ward offers both inpatient and outpatient mental health services alongside epilepsy treatment. More than half (53.5%) of the children and adolescents attending in-patient and/or out-patient mental health clinics carry a diagnosis of epilepsy.

St Joseph’s Rubindi Health Centre III and Mushanga Health center III are Christian faith-based private not for private Health Centers located in Mbarara and Sheema districts respectively. They are headed by medical clinical officers and provide both outpatient and inpatient general medical services to the people from nearby villages. Tukore Invalids Primary School is a government school aided by several Non-government Organizations that support children with special needs by offering them formal primary education by specially-trained teachers. It is located in Mbarara district and receives less privileged children, including refugees, orphans and those with physical and intellectual disabilities from other parts of southwestern Uganda. A team from MRRH, composed of a psychiatrist, post-graduate psychiatry students, a psychiatric clinical officer (psychiatry medical assistant), undergraduate students and a counselor, hold mental health clinics 3 days per month at Rubindi Health Centre III, Mushanga Health Centre III and Tukore Invalids Primary School. These clinics offer assessment, diagnosis and treatment of various psychiatric disorders as well as epilepsy.

### Study population:

we included all children and adolescents aged 5 to 18 years with active epilepsy seeking care from MRRH Psychiatry ward, RHC III, MHC III and TIPS outreach mental health clinics. We defined “active epilepsy” as having had at least one seizure in the past 12 months [16]. A specially trained psychiatry resident, psychiatrist, pediatrician or neurologist confirmed the diagnosis of epilepsy following thorough clinical assessment. Only participants who had a parent/adult caregiver available for the interview were included in the study. Children or adolescents with any emergency medical conditions at the time of the interview were excluded from the study.

### Sample size calculations:

the minimum number of participants required to determine the prevalence of psychiatric disorders and associated factors among children with epilepsy was 140, determined using OpenEpi (online software for sample size calculation for cross sectional studies) [17]. We estimated the prevalence of psychiatric disorders among children and adolescents with epilepsy as 49%, and among children and adolescents without epilepsy as 26%, based on a prior report of these numbers in Kenya [18]. We chose a two-sided significance level (α) = 0.05; and power (1-β,% chance of detecting) = 80%.

### Study variables:

we collected data from study participants about the following variables: age, sex, education level, residence, religion, family history of epilepsy, seizure type, age at seizure onset, use of antiepileptic drugs, use of any other chronic medication, having other chronic illnesses, epilepsy-related physical injuries or deformities and routine use of folic acid by the child or adolescent. These variables were used in the bivariate and multivariate regression analyses described below.

### Ethical considerations:

before starting the data collection process, we obtained written approval of the study from MUST Research Ethical Committee (MUST REC 15/09–17) and Uganda National Council of Science and Technology (SS 4522). This was presented to all the study site administrators for permission to collect data from their centers. Informed consent/+Assent: The research subjects’ participation was voluntary and no incentives were awarded. Participants were informed of the benefits and potential risks of their involvement in the study and that they could opt out at any stage of the study. They were informed of the main elements of the research project so that they could make autonomous decisions as whether to participate or not. Written informed consent was obtained from the parents or the responsible adult care givers and adolescents aged 18 years, while participants below age 18 signed assent forms. Participants were given opportunity to seek clarification on any matter that was unclear to them. Privacy and confidentiality of respondents: respondents were advised about the sensitivity of some questions and were informed that they could choose not to answer if they found some questions difficult or uncomfortable. Interviews were conducted in a private room having only the investigator, participant, and where necessary, the parent/caretaker. Respondents’ confidentiality and integrity were observed. Participants were given unique identification numbers strictly for the purpose of this study and their names, signatures, and those of their relatives were not included in the final report without their consent.

### Participant recruitment:

potential study participants were identified by nurses at MRRH psychiatry ward and by the research assistants at the outreach clinics (RHC III, MHC III and TIPS) using their medical records. Trained psychiatry residents and psychiatrists, in conjunction with a pediatrician or neurologist, further assessed each potential participant with a detailed medical history and physical examination, to confirm the clinical diagnosis of epilepsy. Only those with a clinical diagnosis of epilepsy were recruited for the study after consenting/assenting. Study participants were recruited consecutively until the desired sample size was reached from each study site. They were recruited from MRRH Psychiatry department, RHC III, MHC III and TIPS in the ratio of 2: 1: 1: 1 predetermined by the approximate proportion of epilepsy patients in each of the clinics.

### Study procedures:

participants responded to a piloted investigator-designed questionnaire, which collected data about socio-demographic factors and clinical factors. Their parents/adult guardian responded to the parent version of the Child and Adolescent Symptom Inventory (CASI-5) [19]. CASI-5 is a rating scale for emotional, behavioral and developmental disorders among children and adolescents aged 5–18 years, according to Diagnostic Statistical Manual 5. These disorders include attention deficit hyperactivity disorder, oppositional defiant disorder, conduct disorder, generalized anxiety disorder, social anxiety disorder, separation anxiety disorder, disruptive mood dysregulation disorder, major depressive episode, manic episode, dysthymic disorder, schizophrenia, autistic/Asperger’s disorder, anorexia, bulimia, posttraumatic stress disorder, obsessive-compulsive disorder, specific phobia, panic disorder, selective mutism, trichotillomania, motor tics, vocal tics, and substance use. CASI-5 also assesses the extent to which the symptoms have impaired the social and academic functioning of the child or adolescent. The parent version has 173 items and assesses these disorders as reported by their parents. The adapted version of CASI-5 [20] was translated to Runyankore/Rukiga (local dialect) prior to being used for this study in our setting. Trained research assistants administered the piloted questionnaire using tablets to minimize data loss during transit from the field.

### Statistical analysis:

STATA 14.0 (Stata Corp. LP) was used for statistical analysis. We report population descriptors including prevalence with 95% confidence intervals, and means ± standard deviations. Using CASI-5 scoring [19], we generated symptom cut-off scores based on the number of symptoms necessary for each DSM-5 diagnosis. We also generated impairment cut-off scores for each symptom category, which indicates whether the youth’s social, or academic functioning is impaired by the symptoms. Finally, we generated the clinical cut-off scores for each diagnosis by combining the DSM-5 referenced symptom count cut-off score and impairment cut-off score. We determined the prevalence of each psychiatric disorder, and further grouped these disorders into behavioral, emotional, developmental and psychosis related categories. We initially performed bi-variate logistic regression analyses for each subtype of psychiatric disorder (e.g, behavioral, emotional, developmental, or psychosis related), to determine the association of single clinical variables of interest with each disorder type. We calculated crude odds ratios as a measure of association, with corresponding p-values. We next performed multivariate logistic regression analyses, again using separate models for each of the psychiatric disorder sub-types as above. We included all variables from the bi-variate analysis with a p-value < 0.2, as well as those with clinical relevance from existing literature, in the analysis. Assumption of collinearity was checked by estimating VIF (VIF<10) and tolerance (>20) while outliers were checked by predicting a linear model by estimating dfbetas>0.5 and leverage >0.2. Interaction was assessed using the chunk test, and confounding variables were defined as those with a > 10% difference between the adjusted and crude odds ratios. Goodness of fit was assessed using Hosmer-Lemeshow chi-square test. Variables with p-values less than 0.05 in the multivariate analysis were considered to be significantly associated with the modeled psychiatric disorder type.

## Results

### Study demographics:

our study population consisted of 161 participants with mean age of 12.6 ± 4.4 years. Of these, 102 (63.4%) were from Mbarara district, 21 (13.0%) came from Sheema district while 38 (23.6%) were from other districts. Twenty-three participants (14.3%) reported a family history of epilepsy and 48 (29.8%) had epilepsy-related injuries. Most of the participants 118 (73.3%) were Banyankole, 16 (9.9%) Bakiga, 13 (8.1%) Baganda, while 14 (8.7%) were from other tribes (Table 1). The mean number of seizures experienced by each participant in the prior year was 29.2 ± 99.1, while the mean age at seizure onset was 6.4 ± 4.9 years. Of the 161 participants, 115 (71.4%) were taking antiepileptic medications at the time of the study. 63 (39.1%) reported using carbamazepine, 14 (8.7) phenytoin, 6 (3.7%) phenobarbitone and 6 (3.7%) sodium valproate. 17 (10.6%) reported using both carbamazepine and phenobarbitone, 5 (3.1%) carbamazepine and phenytoin, 3 (1.9%) phenytoin and phenobarbitone while 1 (0.6%) used phenytoin and sodium valproate. 17 (10.6%) of the participants using antiepileptic drugs reported having side effects. 78 (48.4%) participants had used the anti-epileptic drugs (AEDs) for more than 1 year while 37 (23.0%) had used AEDs for less than a year (Table 1).

### Prevalence of emotional, behavioral, developmental and psychosis related disorders:

of the 161 study participants, 93 (57.8%) had at least one psychiatric disorder. Sixty-four (39.8%: 95%CI 32.1 – 47.4) had developmental disorders, 49 (30.4%: 95%CI 23.3–37.6) had emotional disorders, 12 (7.5%: 95%CI 3.4–11.6) had behavioral disorders, while 10 (6.2%: 95%CI 2.4 – 10) had psychosis related disorders (Table 2). The most prevalent emotional disorder was panic disorder (14.3%) while oppositional defiant and conduct disorders were equally prevalent behavioral disorders (6%). Nocturnal enuresis (34.1%) was the most prevalent developmental disorder while schizoid personality was the most prevalent psychosis-related disorder. Eight participants (5.0%) had both emotional and behavioral disorders, 24 (14.9%) had both emotional and developmental disorders, and 7 (4.3%) had both behavioral and developmental disorders.

### Factors associated with psychiatric disorders among children and adolescents with epilepsy:

several socio-demographic and clinical factors were found to be associated with different psychiatric disorders at bivariate analysis (Table 3). Sex, level of education, residence and religion were not associated with any psychiatry disorder category, however, age was significantly associated with emotional, developmental and psychosis related disorders. After controlling for confounders at multivariate analysis, we found that emotional disorders were significantly associated with use of other chronic medication (adjusted odds ratio (aOR) = 3.04, p=0.036) and older age (aOR= 1.13, p=0.007). Developmental disorders were associated with younger age (aOR = 0.86, p=0.001), having epilepsy-related physical injuries (aOR=2.36, p=0.036) and early age at seizure onset (aOR=0.90, p=0.023). Psychosis related disorders were associated with increasing participant’s age (aOR= 1.44, p=0.007), and behavioral disorders had no significant association with any specific factor (Table 4).

## Discussion

This cross-sectional study focused on understanding the psychiatric co-morbidities and associated risk factors in children and adolescents with epilepsy in southwestern Uganda. The prevalence of at least one psychiatric disorder in our study was 57.8% while that of developmental disorders was 39.8%, emotional disorders was 30.4%, behavioral disorders was 7.5% and psychosis related disorders was 6.2%. The prevalence in our study is comparable to 59% found in Swedish community study among children and adolescents with epilepsy and intellectual disability [21]. However, this prevalence is much higher than that reported among children with epilepsy in other parts of the world such as 29.7% in South Carolina, USA [7], 30.3% in Connecticut using clinical assessment [22] and 37% in UK using the development and well-being assessment [23]. This indicates that the risk of having psychiatric disorders among children with epilepsy in southwestern Uganda is higher than that of the developed world. This may have various explanations, including the high level of stigma, misconceptions about epilepsy, poor accessibility to epilepsy treatment, and unaffordable antiepileptic drugs. A combination of these factors may result in late diagnosis and poorly treated epilepsy, hence increasing the risk of psychiatric disorders. Notably though, our reported prevalence was lower than what has been documented in areas such as Brazil (70% among temporal lobe epilepsy (TLE) patients) [24]. However, TLE is known to have a higher association with emotional disorders compared to other types of epilepsy [25]. Generally, the differences in reported prevalence of various psychiatric disorders obtained in various studies may be due to differences in tools used, geographical and socio-cultural settings and study population. This is the first study among children and adolescents in rural Uganda, sub-Saharan Africa that used a DSM-5 based tool to assess epilepsy-related psychiatric comorbidities. The higher prevalence of psychiatric disorders among people with epilepsy in this region compared to those in the developed countries may be due to multiple psychopathologies and other psychosocial stressors such as poverty, poor access to epilepsy treatment as well as stigma and discrimination.

Developmental disorders were the most prevalent in this study (39.8%) and were associated with younger age, having epilepsy-related physical injuries, and early age at seizure onset. This prevalence is comparable to findings in other studies such as 41.7% in Connecticut [22] though in that study, disorders were diagnosed based on clinical assessment and medical records, without any objective assessment tool used. We found that younger children were at increased risk of having developmental disorders. This may be because the risk of brain damage is higher during earlier stages of development. Proper diagnosis and treatment of early childhood epilepsy may be helpful in reducing the risk of developmental disorders in this population. Developmental disorders were also associated with a history of epilepsy-related physical injuries and deformities, which we believe indicates a higher severity of seizure semiology. This could predispose these patients to a greater extent of brain damage, and hence increase the rate of developmental disorders. In developing countries like Uganda, epilepsy has a predominant childhood onset of 6.3 years of age as per this study, and there is a significant delay for families to seek medical intervention. Therefore, continuous untreated or poorly treated seizures may predispose the brain and entire body to damage and lead to various developmental disorders. Elimination disorders (nocturnal enuresis and encopresis) were the most prevalent developmental disorders among these children. This is in line with some studies that also reported possible association between elimination disorders and epilepsy [26–28]. However, it is possible that unrecognized nocturnal seizures may contribute to an increased reporting of bedwetting in the morning. We think this is unlikely to be the case, as prior to data collection, all research assistants were trained on how to differentiate seizure related symptoms (including fecal and urinary incontinence) from psychiatric disorders.

The prevalence of emotional disorders in this study was 30.4% and these were associated with increasing age of the participants. This is higher than what was found in rural north-eastern Uganda where 26.6% of children in the general population were diagnosed with anxiety disorders using the Mini International Neuropsychiatric Interview (MINI) - KID [29]. As children grow, they become more exploratory and want to build more social interactions and bonds. However, repeated seizures may affect their social and self-image, resulting in emotional challenges such as mood changes, anxiety, phobia and other related disorders. This is worsened by the poor societal misconceptions about epilepsy that are characterized by discrimination and stigmatization of these children and adolescents that causes further emotional breakdowns. However, having a positive family history of epilepsy was protective against emotional disorders, suggesting that children born to these families may get some psychological preparation and support from other family members who have some knowledge about epilepsy compared to those with no known family history of the disease. This may reduce the felt stigma and empower the child to cope fairly well with the condition.

Twelve participants (7.5%) had behavioral disorders, which is much lower than what has been documented in other studies such as 49% in Kilifi, Kenya using the child behavioral questionnaire [18], 45% in India using the child behavior checklist [30]. It is also lower than finding of 19.1% in Indonesia where strength and Difficulties Questionnaire was used [31]. This difference may be explained by the difference in tools used in the above studies compared to the one used in this study. These behavioral disorders may be a direct effect of seizures themselves, for example, from post-ictal states or due to underlying brain damage. We did not find a significant association between these behavioral disorders and any socio-demographic or clinical factors, which is different from other studies that found an association with child’s age at seizure onset, etiology and poly-pharmacy [32]. Psychosis related disorders were seen in 6.2% of the participants and these were associated with increasing age. This was comparable to the pooled estimate of 5.6% by Maurice *et al*. [33] from a review of various studies in the developed world. These disorders included schizophrenia and schizoid personality disorder, which are generally chronic disorders with gradual onset. Our study has a number of limitations. First, this was an institutional study, which may not be representative of the exact burden of the psychiatric disorders among children and adolescents with epilepsy in a community setting. Therefore, there is need to conduct broader community based studies to assess the burden of these disorders at a community level and hence develop interventions accordingly.

## Conclusion

This study provides evidence that developmental, emotional, behavioral and psychosis related disorders are highly prevalent among children and adolescents living with epilepsy in southwestern Uganda, and are associated with various socio-demographic and clinical factors. Unfortunately, many of these disorders remain undiagnosed and hence untreated. Therefore, there is need to integrate screening of psychiatric disorders into routine epilepsy care and to treat these disorders concurrently with epilepsy, in order to improve the patients’ outcome as well as quality of life.

## Figures and Tables

**Table 1:** social demographic and clinical characteristic of participants according to the disorders

Characteristic	Behavioural (n=12) n (%)	Emotional (n=49) n (%)	Developmental (n=64) n (%)	Psychosis related (n=10) n (%)	Overall (N=161) n (%)
Age categories					
5–12	9 (75.00)	38 (77.55)	63 (98.44)	9 (90.00)	136 (84.47)
13–18	3 (25.00)	11 (22.45)	1 (1.56)	1 (10.00)	25 (15.53)
Sex					
Male	10 (83.33)	30 (61.22)	40 (62.50)	8 (80.00)	88 (54.66)
Female	2 (16.67)	19 (38.78)	24 (37.50)	2 (20.00)	73 (45.34)
Location					
Urban	0	9 (18.37)	9 (14.06)	2 (20.00)	30 (18.63)
Semi urban	8 (66.67)	18(36.73)	14 (21.88)	3 (30.00)	43 (26.71)
Rural	4 (33.33)	22 (44.90)	41 (64.06)	5 (50.00)	88 (54.66)
Education					
None	7 (58.33)	16 (32.65)	27 (42.19)	4 (40.00)	44 (27.33)
Primary	4 (33.33)	22 (44.90)	34 (53.13)	5 (50.00)	89 (55.28)
Secondary	1 (8.33)	8 (16.33)	2 (3.13)	1 (10.00)	24 (14.91)
Tertiary	0	3 (6.12)	1 (1.56)	0	4 (2.48)
Religion					
Christian	12 (100)	42 (85.71)	60 (93.75)	10 (100)	147 (91.30)
Moslem	0	7 (14.29)	4 (6.25)	0	14 (8.70)
Seizure type					
Generalized	6 (50.00)	37 (75.51)	45 (70.31)	6 (60.00)	121 (75.16)
Focal	6 (50.00)	8 (16.33)	14 (21.88)	2 (20.00)	32 (19.88)
Focal then generalized	0	3 (6.12)	3 (4.69)	1 (10.00)	6 (3.73)
Unknown	0	1 (2.04)	2 (3.13)	1 (10.00)	2 (1.24)
Use of AEDs					
No	8 (66.67)	15 (30.61)	16 (25.00)	4 (40.00)	46 (28.57)
Yes	4 (33.33)	34 (69.39)	48 (75.00)	6 (60.00)	115 (71.43)
Other chronic medication					
No	12 (100)	43 (87.76)	57 (89.06)	9 (90.00)	149 (92.55)
Yes	0	6 (12.24)	7 (10.94)	1 (10.00)	12 (7.45)
Other chronic illness					
No	11 (91.67)	39 (79.59)	49 (76.56)	7 (70.00)	140 (86.96)
Yes	1 (8.33)	10 (20.41)	15 (23.44)	3 (30.00)	21 (13.04)
Use of folic acid					
No	11 (91.67)	35 (71.43)	42 (65.63)	9 (90.00)	104 (64.60)
Yes	1 (8.33)	14 (28.57)	22 (34.38)	(10.00)	57 (35.40)

**Table 2:** proportions of participants with different psychiatric disorders

Disorder	Total number of cases (n=93)	Proportion of all cases (%)
**Emotional disorders**	**(n=49)**	
Selective mutism	3	1.86
Separation anxiety	2	1.24
Social anxiety	1	0.62
Generalized anxiety	4	2.48
Specific phobia	15	9.32
Manic episode	5	3.11
Posttraumatic stress	13	8.07
Panic disorder	23	14.29
Obsessions	15	9.32
Compulsions	12	7.45
Somatic symptoms	15	9.32
Major depressive episode	7	4.35
Persistent depression	2	1.24
Prosocial emotions	5	3.11
Binge eating	4	2.48
Hair pulling	3	1.86
Skin picking	2	1.24
**Behavioral disorders**	**(n=12)**	
Oppositional defiant	6	3.73
Conduct disorder	6	3.73
**Developmental disorders**	**(n=64)**	
ADHD*	18	11.18
Motor tics	8	4.97
Vocal tics	11	6.83
Autism spectrum disorder	13	8.07
Nocturnal enuresis	50	31.06
Encopresis	29	18.01
**Psychosis related disorders**	**(n=10)**	
Schizoid personality	7	4.35
Schizophrenia	6	3.73

ADHD* = Attention deficit hyperactivity disorder

**Table 3:** factors associated with psychiatric disorder categories (bivariate analysis)

Characteristic	Emotional Crude OR (P-value)	Behavioral Crude OR (P-value)	Developmental Crude OR (P-value)	Psychosis related Crude OR (P-value)
Age	1.11 (0.017)	0.95 (0.470)*	0.82 (<0.0001)	1.32 (0.023)
**Age categories**				
5–12	1 (Reference)	1 (Reference)	1 (Reference)	1 (Reference)
13–18	2.03 (0.113)	1.92 (0.354)	0.05 (0.003)	0.59 (0.622)
Sex				
Male	1 (Reference)*	1 (Reference)	1 (Reference)	1 (Reference)
Female	0.68 (0.269)	0.22 (0.056)	0.59 (0.106)	0.28 (0.117)
**Location**				
Urban	1 (Reference)	1 (Reference)	1 (Reference)	1 (Reference)
Semi urban	1.68 (0.304)	4.8 (0.015)	1.13 (0.817)	1.05 (0.959)
Rural	0.78 (0.592)	0.21 (0.015)	2.04 (0.116)	0.84 (0.844)
**Family history of epilepsy**				
No	1 (Reference)*		1 (Reference)	1 (Reference)
Yes	0.3 (0.062)	---	0.62 (0.327)	0.65 (0.691)
**Education level**				
None	1 (Reference)	1 (Reference)	1 (Reference)	1 (Reference)
Primary	0.57 (0.164)	0.25 (0.034)	0.39 (0.013)	0.60 (0.457)
Secondary	0.88 (0.803)	0.23 (0.182)	0.06 (<0.0001)	0.43 (0.468)
Tertiary	5.25 (0.166)	---	0.21 (0.192)	---
Age at seizure onset	1.04 (0.211)*	1.04 (0.518)	0.84 (<0.0001)	0.95 (0.475)
**Seizure type**				
Generalized	1 (Reference)	1 (Reference)	1 (Reference)	1 (Reference)
Focal	0.75 (0.539)	4.42 (0.016)	1.32 (0.498)	1.28 (0.771)
Focal then generalized	2.27 (0.329)	..	1.69 (0.532)	3.83 (0.252)
Unknown	2.27 (0.566)	...	...	19.17 (0.045)
**Use of AEDs**				
No	1 (Reference)	1 (Reference)	1 (Reference)	1 (Reference)
Yes	0.87 (0.705)	0.17 (0.006)	1.34 (0.416)	0.58 (0.414)
**Other chronic medication**				
No	1 (Reference)		1 (Reference)	1 (Reference)
Yes	2.47 (0.136)	...	2.26 (0.181)	1.41 (0.753)
**Other chronic illness**				
No	1 (Reference)	1 (Reference)	1 (Reference)	1 (Reference)
Yes	2.35 (0.072)	0.59 (0.618)	4.64 (0.003)	3.17 (0.116)
**Use of folic acid (by child)**				
No	1 (Reference)	1 (Reference)	1 (Reference)	1 (Reference)
Yes	0.64 (0.232)	0.15 (0.074)	0.93 (0.825)	0.19 (0.118)
**Epilepsy related physical injuries or deformities**				
No	1(reference)	1 (Reference)	1 (Reference)	1 (Reference)
Yes	1.81 (0.102)	2.55 (0.122)	2.99 (0.002)	2.51 (0.161)

**Table 4:** factors associated with psychiatric disorder categories (multivariate analysis)

Characteristic	Emotional			Behavioural			Developmental			Psychosis related		
	AOR	P	C.I	AOR	P	C.I	AOR	P	C.I	AOR	P	C.I
Participant’s age	1.12	0.02	1.02–1.24				0.86	0.002	0.78–0.94	1.44	0.007	1.11–1.87
Age at seizure onset							0.92	0.11	0.83–1.02			
Chronic medical condition												
No(reference)	1						1			1		
Yes	2.61	0.08	0.89–7.67				1.85	0.30	0.58–5.88	5.87	0.068	0.88–39.32
Family history of epilepsy												
No(reference)	1											
Yes	0.22	0.04	0.05–0.95									
Education level												
None(reference)	1						1					
Primary	0.64	0.32	0.27–1.53				0.53	0.16	0.22–1.29			
Secondary	0.62	0.43	0.18–2.06				0.29	0.18	0.05–1.75			
Tertiary	3.65	0.31	0.30–43.09				2.06	0.59	0.15–29.04			
Use of folic acid												
No (reference)				1						1		
Yes				0.39	0.43	0.04–4.02				0.13	0.07	0.01–1.21
Seizure type												
Generalized(reference)				1								
Focal				2.43	0.20	0.62–9.56						
Focal then generalized				--								
Unknown				--								
Sex												
Male(reference)				1								
Female				0.29	0.13	0.06–1.43						
Use of AEDs												
No (reference)				1								
Yes				0.32	0.14	0.07–1.47						
Epilepsy related physical injuries or deformities												
No (reference)				1			1					
Yes				2.5	0.17	0.68–9.23	2.41	0.04	1.06–5.48	1.93	0.39	0.43–8.66

1 (Reference)* indicates variable used in the multiple regression model for that specific category of psychiatric disorders, having a p value > 0.2 but existing literature suggests importance. AOR=adjusted odds ratio, P=p value, C.I= 95% confidence interval

## What is known about this topic

- Epilepsy is highly prevalent among children and adolescents especially in developing countries;
- Children and adolescents with epilepsy are at an increased risk of developing emotional, behavioral, developmental and psychosis-related disorders;
- The psychiatric disorders are associated with varying socio-demographic and clinical factors.

## What this study adds

- The prevalence of at least one psychiatric disorder was 57.8%, with 39.8% of the children and adolescents having developmental disorders, 30.4% having emotional disorders, 7.5% having behavioral disorders, and 6.2% having psychosis related disorders;
- Developmental disorders were associated with young age and having epilepsy-related injuries and deformities, while increasing age was associated with emotional and psychosis related disorders;
- Having a family history of epilepsy was protective against emotional disorders.
